# Microwave-Assisted Preparation of Coffee-Based Activated Carbons: Characteristics, Properties, and Potential Application as Adsorbents for Water Purification

**DOI:** 10.3390/molecules30204123

**Published:** 2025-10-17

**Authors:** Przemysław Pączkowski, Viktoriia Kyshkarova, Viktor Nikolenko, Oksana Arkhipenko, Inna Melnyk, Barbara Gawdzik

**Affiliations:** 1 Department of Polymer Chemistry, Institute of Chemical Sciences, Faculty of Chemistry, Maria Curie-Skłodowska University, Gliniana 33, 20-614 Lublin, Poland; przemyslaw.paczkowski@umcs.pl; 2 Department of Physical and Physico-Chemical Methods of Mineral Processing, Institute of Geotechnics, Slovak Academy of Sciences, Watsonova 45, 040 01 Košice, Slovakia; kyshkarova@saske.sk (V.K.); melnyk@saske.sk (I.M.); 3 Department of Nuclear Physical Technologies, State Institution “The Institute of Environmental Geochemistry of National Academy of Sciences of Ukraine”, Academician Palladin Avenue 34-a, 03142 Kyiv, Ukraine; borey57@gmail.com (V.N.); archipenko@nas.gov.ua (O.A.); 4 Pulsar LLC, Science and Engineering Group Pulsar Limited, Pivnichna 3, 04214 Kyiv, Ukraine; 5 Department of Chemisorption and Hybrid Materials, Chuiko Institute of Surface Chemistry, National Academy of Sciences of Ukraine, Oleha Mudraka 17, 03164 Kyiv, Ukraine

**Keywords:** activated carbon, spent coffee grounds, phosphoric acid activation, microwave-assisted carbonization, cold plasma treatment, plasma-modified carbon, ciprofloxacin

## Abstract

Activated carbons were synthesized from coffee grounds using phosphoric acid as a chemical activator and microwave-assisted carbonization as a rapid and energy-efficient method. Then the prepared carbons were surface-treated with cold plasma to improve their chemical properties and adsorption efficiency. The structural properties and chemical structure of the carbons were determined using nitrogen adsorption–desorption analysis, X-ray photoelectron spectroscopy, as well as X-ray microanalysis by means of scanning electron microscopy. The effect of cold plasma treatment on surface functionality and porosity was investigated. The resulting activated carbons were tested for their potential use as sorbents for the removal of ciprofloxacin, a commonly used antibiotic, from aqueous solutions. The effects of solution pH, sorption kinetics, and initial concentration were investigated. Adsorption kinetics followed a pseudo-second-order model, and the equilibrium data were well described by both the Langmuir and Freundlich isotherms, indicating a combination of monolayer adsorption on homogeneous sites and multilayer adsorption on heterogeneous surfaces. Plasma-treated carbon demonstrated significantly increased adsorption capacity (42.6–120.6 mg g^−1^) compared to the unactivated samples (20.2–92.4 mg g^−1^). Desorption experiments revealed that the plasma-treated carbon retained over 90% efficiency after seven cycles, confirming its excellent reusability and regeneration potential for practical water treatment applications.

## 1. Introduction

Agricultural wastes such as peanut husks, wood sawdust, rice husks, and corn stover are commonly used as adsorbents for the removal of various pollutants from water. Coffee is a crop, and coffee waste is a promising green, low-cost, and renewable wastewater treatment matter that can be used as a bio-based adsorbent to remove pollutants [1].

Coffee plants belong to the Rubiaceae family. The plant species *Coffea arabica* (Arabica) and *Coffea canephora* (Robusta) are the most popular and account for 75 and 25% of the world’s coffee production for drinking purposes, respectively [2,3].

According to the International Coffee Organization (ICO) statistics, the harvest of coffee beans reached 10.5 million tons in 2020–2021 [4]. Every ton of prepared coffee will produce 0.5 tons of coffee husks, and about 6 million tons of coffee grounds are produced globally each year [5]. These wastes contain some organic compounds, such as caffeine, tannins, and chlorogenic acid which can pollute the environment without proper treatment [6]. Coffee waste also contains large amounts of lipids, cellulose and hemicellulose, polyphenols, carbohydrates, proteins, and various other components that can be converted into valuable products such as biofuels, biochar, dietary fibre, flavours, bioactive compounds, and carotenoids.

Coffee is one of the most common and most frequently consumed beverages in the world. Coffee drinks are considered to possess stimulating and refreshing properties [7]. The main by-products of the coffee industry are spent coffee grounds (SCG), silverskin (CS), and coffee shells (CH).

CH is the epidermis of the coffee beans that falls off during roasting due to the application of heat, while CS is the only by-product of the coffee roasting process. Coffee silverskin is the thin skin on the outer layer of coffee beans—a by-product of the coffee roasting industry. The silver husk is the outer layer of coffee beans, so it can be assumed that at least some of the bioactive compounds present in roasted coffee beans will also remain in the husk. Roasted coffee beans are a rich source of bioactive compounds such as caffeine, chlorogenic acids, caffeic acid, coumaric acid, ferulic acid, protocatechuic acid, vanillic acid, gallic acid, and flavonoids [8]. The content of polyphenolic compounds in the silver husk can be as great as 1600 mg∙100 g^−1^ husk. Due to their presence, this residue is characterized by great antioxidant properties and a large concentration of soluble dietary fibre (86% of the total dietary fibre). In addition to polyphenolic compounds, important bioactive compounds present in coffee and silver husk are caffeine (1,3,7-trimethylxanthine) and some mono- and polysaccharides: cellulose and hemicellulose, xylose, galactose, mannose, arabinose, and glucose, being found in major amounts [9].

In recent years, some studies have also proven the good adsorption action of coffee waste for the removal of organic and inorganic pollutants from wastewater, so it can be used as an adsorbent in wastewater treatment. Spent coffee grounds (SCGs) represent valuable precursors for activated carbon preparation [10,11,12,13,14]. Sorbents obtained from coffee are mainly used for heavy metals and dyes [15].

Jutakridsada et al. [16] impregnated SCG in 60 mL of ZnCl_2_ with various concentrations (5, 10, 15 wt.%), and carbonized it in a furnace with atmospheric air at 400, 450, and 500 °C and used for Cu(II) sorption.

SCG activation using phosphoric acid (H_3_PO_4_) and phosphorus pentoxide for Cu(II) sorption was described in [17].

Sorption of aniline yellow dye was studied on carbon sorbents obtained from SCG soaked in potassium hydroxide (KOH) and carbonized at 500 °C for 30 min in a muffle furnace [18].

Activated carbon obtained from ground coffee, impregnated with 25% *v*/*v* HNO_3_ and activated at 500 °C for 20 min was used for the sorption of methyl orange [19].

Activated carbon from SCGs [20], provided by an Italian local cafeteria (100% Arabica), was mixed with potassium hydroxide (KOH) powder at the 1:1 mass ratio. KOH is commonly used as an activating agent to develop porosity during thermal treatments and was pyrolyzed in a tubular alumina reactor (Carbolite) at 800 °C for 4 h under N_2_ atmosphere in the sorption of dyes (methylene blue (MB), erythrosine B (EB) and bromothymol blue) as well as phenols (3-chlorophenol and bisphenol-A).

Block et al. [21] described the preparation of carbon adsorbents from spent coffee for the removal of methylene blue and methyl orange from water.

Chemical activation of carbons prepared from coffee residue was reported in [22], while their use as CO_2_ capture adsorbents was described in [23].

In all the cases mentioned above, carbonization was carried out in a long process in traditional furnaces. Recently, attempts have been made to obtain carbon sorbents from coffee waste using a microwave treatment process of biomass [24]. To obtain carbon from coffee ground waste, Kang et al. [25] used an N_2_ plasma jet for the carbonization process and a CO_2_ plasma jet for the activation process.

In this paper, carbon sorbents obtained from coffee residues carbonized under the influence of microwave radiation and activated in a plasma reactor are presented. All samples were obtained after earlier impregnation by H_3_PO_4_. The influence of carbonization time on chemical and porous structure of the obtained carbons was studied. Comparative studies of antibiotic sorption on carbon sorbents obtained during microwave irradiation and on sorbents activated additionally with plasma were also carried out.

## 2. Results and Discussion

The spent coffee grounds used in the experiments were subjected to the characterization of their porous structure. The specific surface area was approx. 10 m^2^ g^−1^ and the total pore volume approx. 0.01 cm^3^ g^−1^.

The results of the porous structure determination for carbons are presented in Table 1. The samples C2, C5, and C10, obtained from SCGs during irradiation of microwaves, are porous. As the microwave exposure time increases, a porous structure develops. The specific surface area of the samples increases from 26 m^2^ g^−1^ for the sample irradiated for 2 min to 482 m^2^ g^−1^ for the sample exposed to microwaves for 10 min. At the same time, the pore diameters decrease, and for the samples exposed to microwaves for 5 and 10 min, micropores appear. This phenomenon is clearly visible from pore size distribution, showing two-modal distributions of pore sizes (desorption branch).

This indicates that as the microwave exposure time increases, the structure of the forming carbons undergoes a reorganization. Microwave irradiation enabled the rapid internal heating of the samples, facilitating the release of gaseous products and pore development in a short time. It is assumed that graphitization progresses in their structure.

The porous structure of samples additionally activated by plasma has become more developed. Applying cold plasma to the samples increased their pore volume and specific surface areas. For the samples C5 and C10, the specific surface areas increased after plasma activation, from 325 and 482 m^2^ g^−1^ to 425 and 601 m^2^ g^−1^, respectively. Even the carbon obtained after 2 min of carbonization, which had a specific surface area of only 26 m^2^ g^−1^, increased to 44 m^2^ g^−1^ after plasma activation, and the pore volume increased from 0.108 cm^3^ g^−1^ to 0.155 cm^3^ g^−1^.

The results for this sample demonstrated that plasma alone is unlikely to induce significant porosity in slightly carbonized structures and should be used as a complementary surface treatment method.

**Figure 1:**
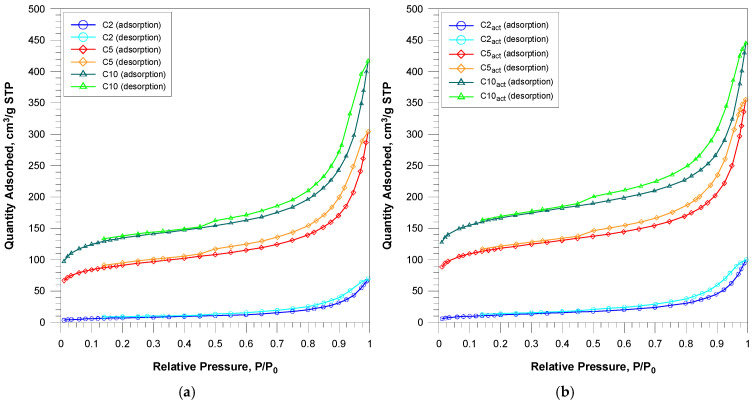
Nitrogen adsorption–desorption isotherms of coffee-based activated carbons, before (**a**) and after (**b**) plasma treatment.

The nitrogen adsorption/desorption curves are presented in Figure 1. Nitrogen adsorption–desorption isotherms for the obtained carbons can be assigned to the IUPAC type IV classification. Capillary condensation occurs with an accompanying H3/H4 type hysteresis loop. The isotherms for all materials are typical for the studied carbons with a mixed micro-/mesoporous structure.

**Figure 2:**
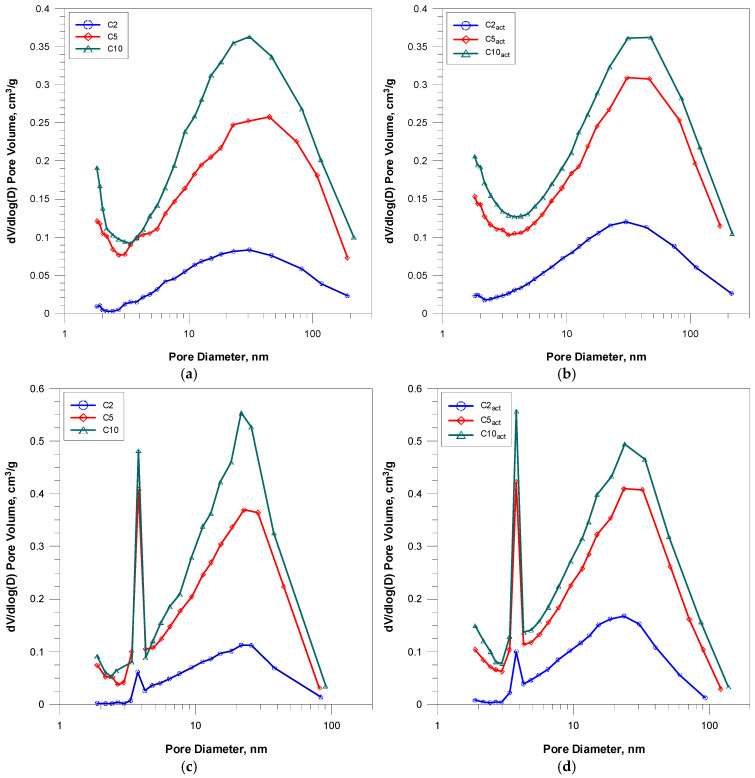
Pore size distributions (PSDs) of coffee-based activated carbons: from adsorption branch (**a**,**b**) and desorption branch (**c**,**d**).

Based on their courses, pore size distribution curves were determined (Figure 2). The curves a and b in Figure 2, relating to the starting carbon materials and their cold plasma-activated counterparts, are derived from the nitrogen absorption branch. The curves in Figure 2c,d originate from the desorption branch. Based on their course, their most probable pore diameters can be determined. For the curves obtained from the adsorption branch for the samples before and after activation, the pore size distribution does not change significantly, and the most probable pore diameter is 30 nm [26]. The PSD curves from the desorption branch show a bimodal pore distribution with the maxima around 4 and 22 nm, both for the samples before and after plasma activation.

The results from the SEM-EDS microanalysis show that with the increase in the time of microwave exposure to spent coffee impregnated with H_3_PO_4_, its carbonization occurs (Table 2). The percentage of carbon for sample C2, which is 62.54%, increases to 77.37% for the sample irradiated for 10 min. At the same time, a large decrease in the percentage of oxygen can be observed. High temperature causes the breakdown of chemical bonds present in coffee, resulting in the release of volatile oxygen-containing compounds. The content of nitrogen in the studied carbon sorbents’ structures decreases relatively slowly. In turn, the percentage of phosphorus increases insignificantly. This indicates that the phosphoric acid used to impregnate the spent coffee was transformed into more stable compounds and it remained in this form in the carbonization products.

Similar results were obtained for the samples additionally activated with cold plasma. Differences in the content of individual elements did not exceed 0.5–1%. The largest differences can be observed for oxygen and phosphorus. For these elements, the percentage content increases insignificantly after plasma activation.

The results show that plasma activation induced very limited changes in elemental composition, as evidenced by the close similarity of each sample to its plasma-treated counterpart. These minimal differences suggest that plasma treatment affects primarily the surface chemistry, as confirmed by the XPS analysis, without significant alteration of the chemical composition. This is to be expected, as cold plasma operates at low temperatures and shallow penetration depths, making it an effective tool for surface functionalization without damaging the internal structure of carbon sorbents.

**Figure 3:**
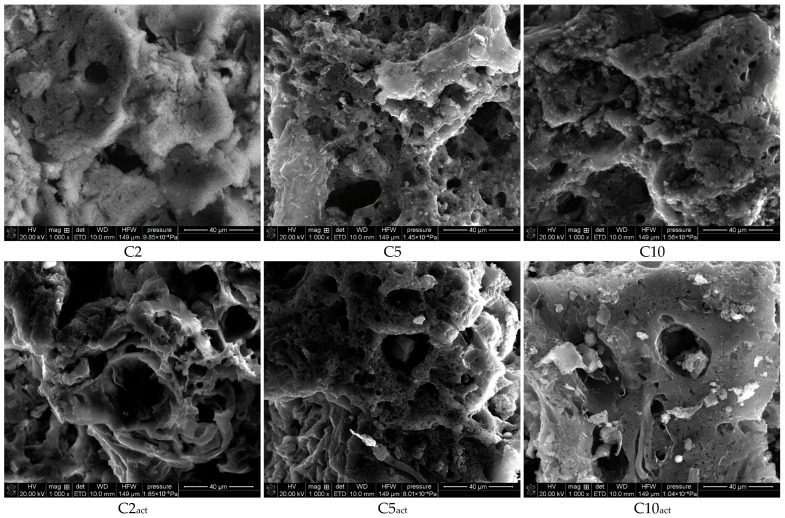
SEM images of activated carbons at 1000× magnification.

Figure 3 presents the SEM images of the obtained carbons at 1000× magnification. The photographs confirm the developed porous structure of the obtained carbons. Small clusters can be observed on the surface of the samples activated by plasma.

Detailed X-ray photoelectron spectroscopy (XPS) studies were carried out to elucidate their origin. This technique allows also for the tracking of changes in the composition of the resulting carbon sorbents with the increasing microwave irradiation time and subsequent plasma activation.

Surface elemental composition and chemical bonding types present in the coffee-based activated carbons were investigated using the XPS technique. The evolution of the carbon (C 1s), oxygen (O 1s), nitrogen (N 1s), and phosphorus (P 2p) functional groups following phosphoric acid activation, followed by microwave irradiation (2, 5, and 10 min) and subsequent cold plasma treatment, was examined. High-resolution deconvolution of the spectra revealed significant transformations of the surface functional groups.

**Figure 4:**
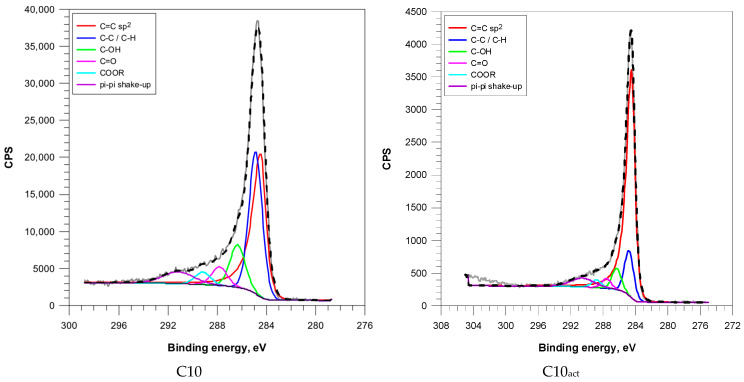
C (1s) XPS spectra of coffee-based activated carbons.

The C 1s peak of the XPS spectra (Figure 4 and Figure S1) was deconvoluted into six main components: sp^2^-hybridized carbon (C=C), saturated carbon (C–C/C–H), hydroxyl/ether groups (C–OH), carbonyl groups (C=O), carboxyl/ester functionalities (COOR), and π–π* shake-up.

Microwave irradiation induced progressive aromatization of the carbon matrix. The content of sp^2^ carbon increased from 41.2% (C2) to 60.2% (C10), suggesting extended formation of graphitic domains (Table 3).

Cold plasma treatment resulted in a significant increase in sp^2^-hybridized carbon across all samples, as well as a significant reduction in aliphatic and hydroxyl species. This indicates to efficient removal of disordered carbon and enhancement of graphitic order. Interestingly, C2_act_ retained elevated C=O (11.3%) and moderate C–OH (9.3%) levels, suggesting that plasma treatment of less aromatized surfaces favours the retention or reintroduction of oxygenated groups.

**Figure 5:**
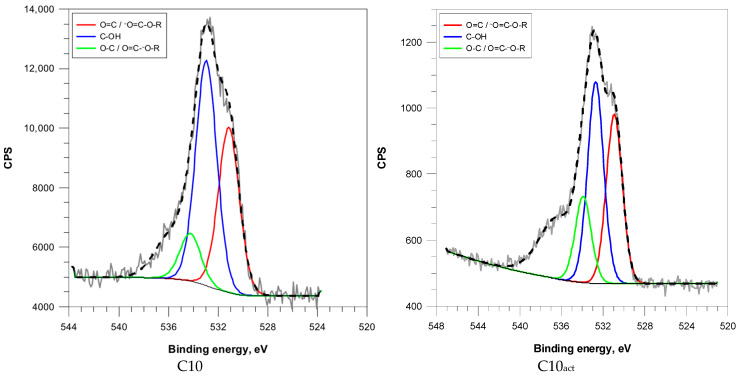
O (1s) XPS spectra of coffee-based activated carbons.

Deconvolution of the O 1s peak spectra identified four types of oxygen functionalities: carbonyl/carboxyl oxygen (O=C/O=C–O–R), hydroxyl groups (C–OH), and ether/phenolic groups (C–O/O–C=O) (Figure 5).

The increasing microwave duration correlated with a significant enrichment in carbonyl functionalities—from 18.1% in C2 to 38.8% in C10—and a corresponding decline in the hydroxyl content (Figure 5 and Figure S2). This trend indicates thermal oxidation and the rearrangement of oxygenated species during the microwave treatment. The decrease in ether/phenolic oxygen indicates further decomposition of labile oxygen functionalities for both activated and nonactivated samples. Comparing the concentrations of oxygen functional groups in samples before and after plasma activation, an increase can be observed. Similar observations regarding the increase in the presence of these functional groups in carbon exposed to non-thermal plasma were made by Zhang et al. [27].

C2_act_ exhibited a large ether/phenolic content (30.5%) and moderate carbonyl content (20.4%), suggesting effective oxidative functionalization of a less graphitized surface. C5_act_ and C10_act_ showed elevated carbonyl contents (32.1% and 42.0%, respectively), probably due to selective oxidation of sp^2^-rich domains. (Table 4).

**Figure 6:**
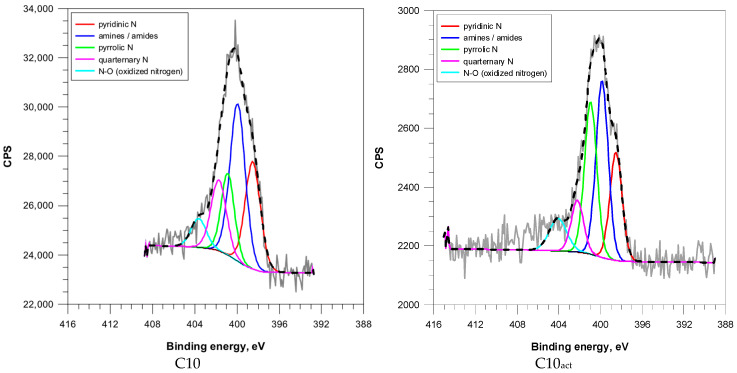
N (1s) XPS spectra of coffee-based activated carbons.

According to Figure 6 and Figure S3, the N 1s peak spectra revealed five distinct nitrogen species: pyridinic N, pyrrolic N, graphitic (quaternary) N, amines/amides, and oxidized nitrogen (N–O).

In the samples treated solely by microwave irradiation, a progressive transformation of amines/amides (from 57.3% in C2 to 36.2% in C10) into pyridinic and graphitic nitrogen species was evident (Table 5). This indicates the increasing incorporation of nitrogen into aromatic structures through cyclization, enhancing nitrogen stability.

C2_act_ exhibited the largest concentration of pyrrolic N (38.1%) and a significant concentration of quaternary N (22.6%) as well as a significant reduction in amines/amides (15.3%), suggesting strong plasma dehydrogenation and nitrogen heterocycle formation. C5_act_ and C10_act_ retained a balanced distribution of pyridinic, pyrrolic, and graphitic nitrogen, reflecting a gradual transformation due to their aromatized nature.

Pharmaceuticals are increasingly recognized as emerging contaminants in water resources. A wide range of treatment strategies have been investigated to remove them, including conventional methods such as biodegradation, adsorption, and activated sludge as well as advanced techniques like membranes, microfiltration, and ozonation [28]. Among these, activated carbon has proved particularly effective for antibiotic removal due to its large specific surface area, large porosity, and favourable pore size distribution [29].

**Figure 7:**
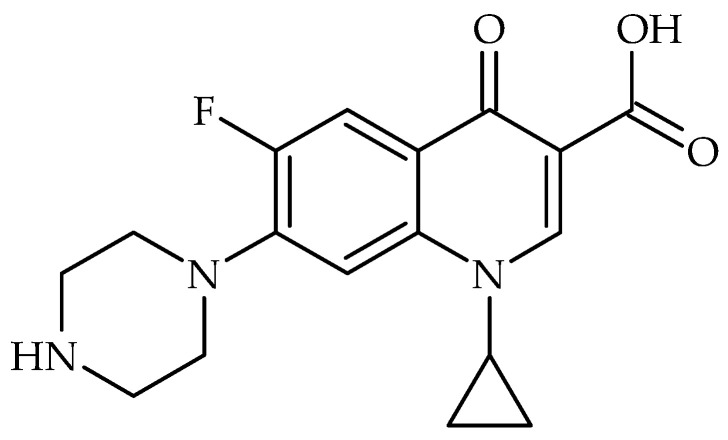
Ciprofloxacin (1-cyclopropyl-6-fluoro-4-oxo-7-(piperazin-1-yl)-1,4-dihydroquinoline-3-carboxylic acid) structure.

All the studied carbons were used in purification water investigations. As a test compound—antibiotic ciprofloxacin was chosen (Figure 7). According to Balarak et al., the ciprofloxacin molecule has the following dimensions: length—1.31 nm; width—0.82 nm; and height—0.25 nm [30].

**Figure 8:**
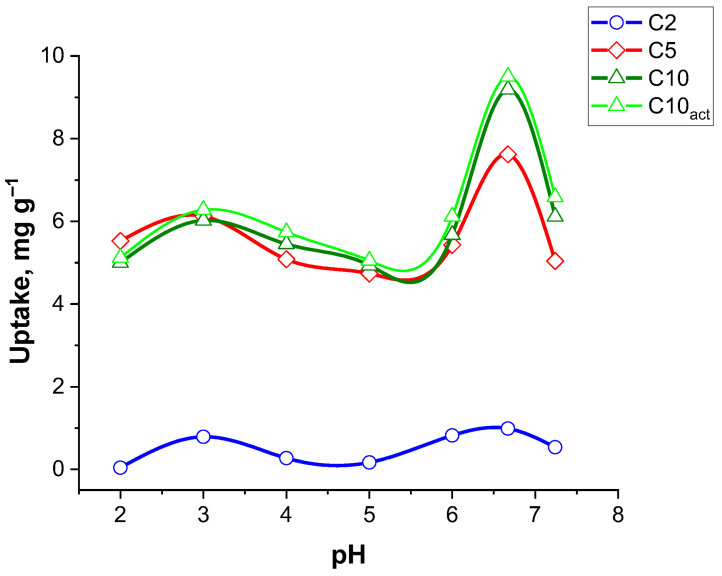
Investigation of the pH effect on adsorption of CF for carbonized samples.

As a first step, we assessed how solution pH governs ciprofloxacin (CF) adsorption on the carbon adsorbents. Figure 8 illustrates the effect of pH on the adsorption capacity of CF for three different adsorbent samples: C2, C5, C10, and C10_act_. The uptake capacity is measured in mg g^−1^ across a pH range of 2 to 7.5.

Samples C5, C10, and C10_act_ exhibit significantly greater adsorption capacities compared to C2 across the entire pH range, with C10_act_ showing the highest uptake overall. C5, C10, and C10_act_ display relatively stable adsorption from pH 2 to around pH 6, with the uptake values ranging from approximately 5 to 6 mg g^−1^. A sharp increase in adsorption is observed as the pH approaches neutral (pH ~7), where C10_act_ reaches a maximum uptake of ~9.5 mg g^−1^, while C5 peaks slightly lower at ~7 mg g^−1^.

This pH-dependent behaviour suggests that adsorption is more favourable at near-neutral pH, likely due to the deprotonation of functional groups on the adsorbent surface which enhances interactions with the ciprofloxacin molecules, a trend that has also been reported in previous studies [31,32]. Additionally, the increase can correspond to a reduction in competition between hydrogen ions and CF for active groups (see ciprofloxacin structure Figure 7).

**Figure 9:**
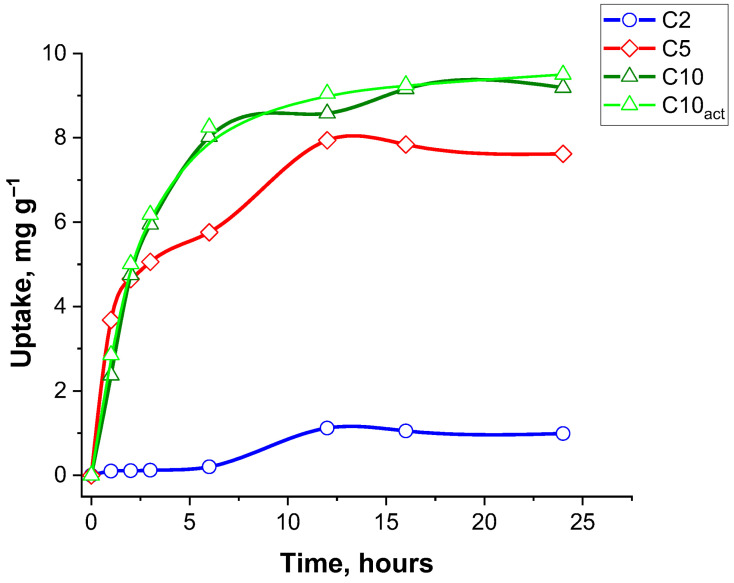
CF adsorption kinetic curves for the carbonized samples.

Figure 9 presents the adsorption kinetics of ciprofloxacin by four adsorbent samples—C2, C5, C10, and C10_act_—over a 24 h period. Adsorption capacity (mg g^−1^) is plotted against the contact time (hours), providing insight into the rate and extent of CF uptake by each carbon material.

Samples C10 and C10_act_ exhibit the largest adsorption capacity among all samples, reaching a plateau of approximately 9.5 mg g^−1^. The uptake increases rapidly during the first 3 h, indicating a high initial adsorption rate due to the abundance of available active groups. After about 6 h, the curve begins to stabilize, suggesting that equilibrium is reached between 12 and 15 h. This rapid and high-capacity adsorption can be attributed not only to the specific surface area and well-developed porous structure of the material, which promote efficient diffusion and accessibility of active sites for ciprofloxacin molecules, but also to the presence of surface functional groups. These groups facilitate specific interactions, such as hydrogen bonding and electrostatic attraction, enhancing the affinity between the adsorbent and the antibiotic. Moreover, the activation process may increase the availability or reactivity of these functional groups, strengthening their ability to form hydrogen bonds and thus further contributing to the overall adsorption performance.

The C5 adsorbent shows a similar kinetic trend, though with a slightly smaller adsorption capacity, peaking around 7.5 mg g^−1^.

In contrast, the carbon sample C2 demonstrates a very small uptake capacity, reaching only about 1 mg g^−1^ after 24 h. The adsorption process is also significantly slower, with only marginal increases in uptake over time.

Table 6 presents the kinetic parameters for the adsorption of CF (ciprofloxacin) onto the samples C2, C5, C10, and C10_act_, derived from both pseudo-first-order and pseudo-second-order kinetic (PSO) models. The experimental adsorption capacities (q_e_ (exp)) ranged from 1.12 to 9.50 mg g^−1^, increasing for the samples C2 to C10_act_. For the pseudo-first-order model, the calculated equilibrium adsorption capacities (q_e_ (cal)) deviated significantly from the experimental values, and the coefficients of determination (R^2^) were relatively small (0.047–0.867), indicating the poor fit of the model.

In contrast, the pseudo-second-order model provided much better agreement with the experimental data. The q_e_ (cal) values closely matched the q_e_ (exp), and high R^2^ values (0.994–0.998) were observed across all samples. Notably, the rate constant k_2_ was the highest for the sample C10_act_ (0.251 g mg^−1^ h^−1^), indicating a faster sorption process at lower adsorption capacity. Overall, the pseudo-second-order model more accurately represents the kinetic behaviour of ciprofloxacin adsorption onto the tested materials. This agreement indicates a surface-reaction-controlled process; adsorption rates are governed by the availability of active sites and specific interactions between CF and carbon surface groups (electrostatic complexation/H-bonding and possible π–π* interactions), rather than by intraparticle diffusion alone. The observed PSO kinetics indicate a chemisorption-like, site-limited uptake, in which adsorption rates are governed by surface functionality and the pH-dependent speciation of CF.

**Figure 10:**
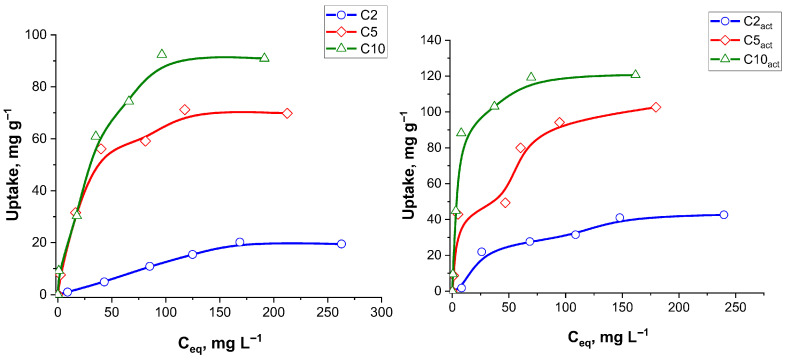
Isotherms of CF adsorption on the initial and plasma-activated carbon samples.

Isotherms of ciprofloxacin adsorption on the initial and plasma-activated carbon samples are presented in Figure 10. Table 7 summarizes the adsorption isotherm parameters for ciprofloxacin onto various adsorbent samples, evaluated using both the Langmuir and Freundlich models. The maximum monolayer adsorption capacities (q_max_) estimated from the Langmuir model ranged from 30.3 to 138.9 mg g^−1^, increasing with sample activation and suggesting enhanced surface area or active groups accessibility upon plasma treatment. The Langmuir model demonstrated excellent fitting for all samples, with the R^2^ values exceeding 0.994 in most cases, indicating that monolayer adsorption on a homogenous surface is a dominant mechanism.

The Freundlich model also yielded reasonable fits, particularly for C2 (R^2^ = 0.972) and C5_act_ (R^2^ = 0.977), though in general, the R^2^ values were smaller than those of the Langmuir model. The Freundlich constants 1/*n* ranged from 0.45 to 1.01, indicating favourable adsorption across all samples (0 < 1/*n* < 1), with C2_act_ exhibiting a nearly linear sorption process (1/*n* = 1.01). The Freundlich adsorption capacity parameter (K_F_) peaked for C10_act_ at 18.35 mg g^−1^, consistent with its high q_max_ in the Langmuir model.

Overall, the data indicate that the Langmuir model provides a better representation of the adsorption process for ciprofloxacin, particularly after the sample treatment with plasma, highlighting the role of monolayer adsorption on the well-defined active sites. Importantly, the good fit of both the Langmuir and Freundlich models indicates that the adsorption process is complex, involving both monolayer adsorption on uniform sites and multilayer adsorption on heterogeneous surfaces. This dual compatibility suggests that ciprofloxacin interacts with the carbon surface through multiple mechanisms, including specific binding to active sites and non-specific interactions across varied surface regions. To assess the adsorption performance of C10_act_ for ciprofloxacin removal, its maximum adsorption capacity was compared with that of previously reported adsorbents. A summary of the comparative data is provided in Table 8.

**Figure 11:**
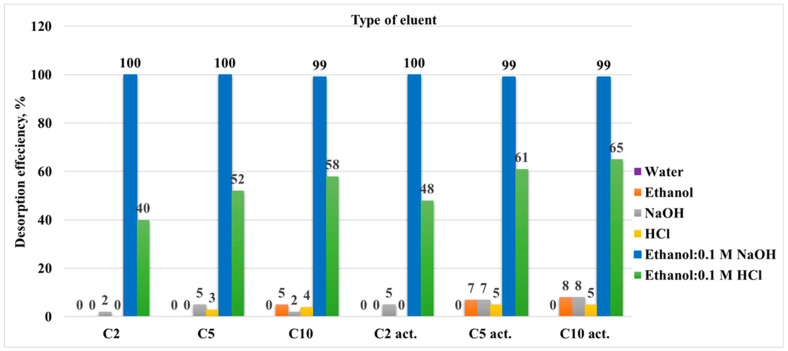
Effect of eluent type on the desorption efficiency of ciprofloxacin.

Desorption studies are essential to evaluate the reusability of the adsorbent and to understand the strength and reversibility of the adsorbate-adsorbent interactions. The desorption results for each sample, depending on the eluent used in the first desorption cycle, are presented as a bar chart in Figure 11. The data show that, regardless of the sample, the desorption behaviour for each eluent is consistent with the ethanol–alkali mixture (70:30) EtOH: 0.1 M NaOH showing the largest desorption efficiency.

This observation is not unexpected and supports the proposed adsorption mechanism. Since ciprofloxacin hydrochloride was adsorbed onto the sample surface, and the adsorption probably involved both hydrophobic and electrostatic interactions, the desorption process is most effective when using ethanol (disrupting hydrophobic interactions) and alkali (disrupting electrostatic interactions).

For testing the eluents and comparing the samples, all samples were used in this procedure. However, only the sample C10_act_ that exhibited the best adsorption activity was used to study 10 adsorption–desorption cycles under the selected optimal conditions (adsorption from the aqueous solution, desorption using the ethanol–alkali mixture).

**Figure 12:**
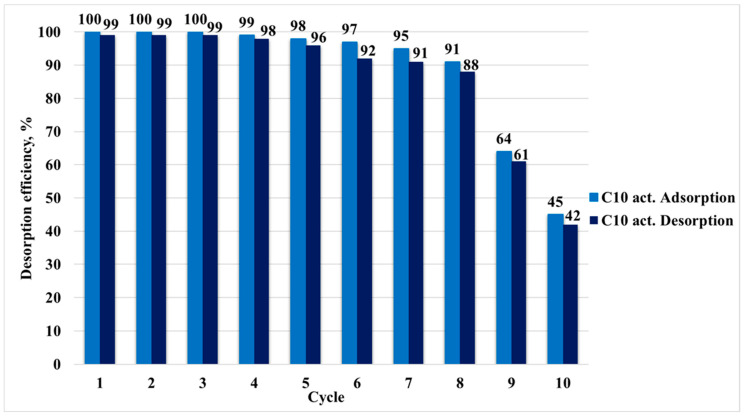
Adsorption–desorption cycles of ciprofloxacin on the plasma-activated sample C10.

Figure 12 illustrates the cyclic adsorption and desorption efficiencies of CF onto the plasma-activated C10_act_ sample over ten consecutive regeneration cycles. The initial cycles (1–4) demonstrate nearly complete adsorption (100%) and desorption (99%) efficiencies, indicating the excellent regeneration performance and chemical stability of the adsorbent. This effective performance suggests that the C10_act_ remains largely unaffected in the early reuse stages.

However, a gradual decline in both adsorption and desorption efficiencies began at cycle 5. By cycle 6, adsorption had decreased to 97% and desorption to 92%, indicating the onset of slight degradation of active sites or incomplete desorption of ciprofloxacin. The decrease became pronounced from cycles 7 to 10, with adsorption dropping from 95% to 45% and desorption from 91% to 42% [41]. Regeneration was performed with a NaOH/ethanol mixture. As reported for organic pollutants, alkaline desorbents typically outperform acidic ones due to electrostatic repulsion at elevated pH and generally cause minimal surface damage; nevertheless, the sharp deterioration beyond cycle 8 is best explained by progressive alteration/depletion of plasma-generated surface functional groups (e.g., carboxyl and amino) and partial pore blockage by residual organics not fully removed by NaOH/ethanol (ethanol disrupts hydrophobic and hydrogen bond interactions but may not eliminate strongly bound species).

**Figure 13:**
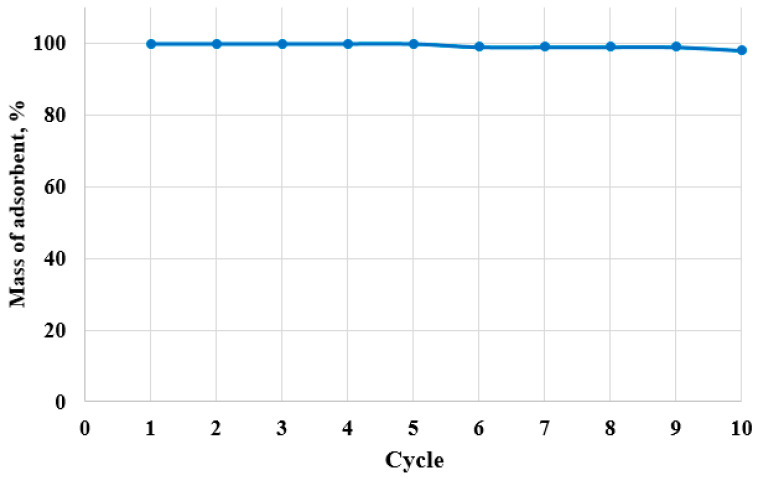
Stability of adsorbent (C10_act_) mass over multiple adsorption–desorption cycles.

Loss of adsorbent material was ruled out. No visible physical degradation was observed over ten adsorption–desorption cycles, and the relative mass after each cycle remained essentially constant (Figure 13), confirming physical stability and implicating surface chemical changes and fouling, rather than material loss, as the main causes of performance decline.

Despite this decline, C10_act_ maintains reasonably great performance up to the 7th cycle (>90% efficiency), underscoring its good reusability and stability for practical applications. Nonetheless, a sharp decrease beyond cycle 8 suggests the need for reactivation treatment or replacement after a certain number of cycles to maintain effective performance.

Carbons obtained from coffee grounds are capable of specific sorption of ciprofloxacin. The sorption mechanism is based, among others, on interactions between ciprofloxacin molecules and aromatic carbon rings, as well as proton donor and electrostatic interactions of the sorbents with functional groups present in the pesticide’s structure. These assumptions are confirmed by XPS results indicating the formation of a surface rich in oxygen-containing functional groups with increasing carbonization time in a microwave reactor. This effect is most evident in the C10_act_ sample, which was additionally enriched in oxygen-containing carbonyl groups through plasma activation.

## 3. Materials and Methods

### 3.1. Materials

Spent coffee grounds (100% Arabica) were used as precursors for the preparation of carbon sorbents.

Phosphoric acid (H_3_PO_4_) provided by POCh (Gliwice, Poland) was used as a spent coffee grounds chemical activator responsible for formation of porous structure of carbon materials. It also plays a role as a burning protector during the microwave irradiation.

Ciprofloxacin hydrochloride monohydrate (Thermo Scientific Chemicals, Ward Hill, MA, USA) was used in the sorption studies.

Ethanol, NaCl, HCl, and NaOH from ITES (Vranov, Slovakia) were used in the ciprofloxacin ad-/desorption experiments.

### 3.2. Preparation of Sorbents by Microwave Irradiation

Carbon sorbents were obtained by microwave-assisted carbonization of SCGs with the presence of phosphoric acid at 2, 5, and 10 min. In the following text, these samples will be referred to as C2, C5, and C10. The coffee residues were impregnated with 85% phosphoric acid at the impregnation ratio of 1:1, and then carbonized in a microwave reactor. This acid acts also as an effective microwave absorber. The microwave irradiation was performed using the MAS-II Plus Microwave Synthesis Workstation from SINEO Microwave Chemistry Technology Co., Ltd. (Shanghai, China). The samples were exposed to microwave radiation at a power of 1000 W, which is sufficient power to create a pore structure in the heated [42,43]. The produced sorbents were washed in the Soxhlet apparatus with distilled water until pH 7 was obtained. The spent coffee ground without phosphoric acid was used as a reference, but it burned out completely in the microwave reactor.

### 3.3. Activation of Sorbent Surfaces with Plasma

For the activation of carbon sorbent surfaces, a cold plasma NTP reactor designed by Pulsar Inc. (Kyiv, Ukraine) was used. The parameters of the plasma generator were as follows: voltage—6–50 kV, DC; pulse frequency—10–100 kHz; current—1–50 A; power—1–3.0 kW; distance between electrodes—5–100 mm; input—220 V; and alternating current (AC)—1–15 A.

The studied materials were exposed to cold plasma in the form of aqueous suspension. Carbon adsorbents were processed under the following conditions: the electrode voltage—8 kV; the pulse frequency—63 kHz; the pulse duration—5 μs. The installation capacity was 1 L/10 s.

The procedure for all the studied samples (C2, C5, C10) was the same. A measure of 5 L of distilled water was added to 0.75 g of carbon. The initial temperature was 14 °C. Stage I—the processing time 100 s (2 passes); Stage II—the processing time 100 s (2 passes). The final temperature was 22 °C.

After processing the samples were in the form of suspensions. Carbons obtained in this process are called C2_act_, C5_act_, and C10_act_.

### 3.4. Chemical Structure Analysis

The chemical structure of the obtained carbons was studied by SEM-EDS and photoelectron microscopy EDX analyses using Quanta 3D FEG Microscopy with an EDX detector, FEI (Hillsboro, OR, USA). SEM EDX analysis combines Scanning Electron Microscopy (SEM) to provide high-resolution images of sample surfaces with an Energy Dispersive X-ray Spectrometer EDX Octane Elect Plus (Berwyn, IL, USA) to identify and quantify elemental compositions. When the sample is bombarded with an electron beam in SEM, it emits characteristic X-rays detected by EDX, allowing for simultaneous morphological and chemical analyses in the micro- to nanoscales. The test was carried out at an accelerating voltage of 20 kV. The samples were analyzed in five replicates. The obtained values were presented as the weight percentage (wt.%).

XPS spectra were obtained using an ultra-high vacuum (<2 × 10^−8^ Pa) multi-chamber UHV system (Prevac, Poland) equipped with the Al Kα line excitation source VG Scienta SAX 100 (12 kV, 30 mA), monochromator VG Scienta XM 780, and hemispherical analyzer Scienta R4000 (VG Scienta AB, Uppsala, Sweden). The pass energy and energy steps were 200 eV and 0.5 eV in the survey spectra and 50 eV and 0.1 eV in the detailed spectra. The spectra were calibrated for carbon C 1s excitation at a binding energy of 284.7 eV.

To determine the contribution of sp^2^ carbon photoemission, spectra in the range of 1180–1260 eV (BE) were collected. For determination of the ratio sp^2^/sp^3^, a method from the first derivative of Auger CKLL spectrum was employed [44]. After smoothing and differentiating the spectra, the distance (eV) between the maximum and minimum so-called D-parameter was determined, which indicates how large the contribution of sp^2^ carbon is in the surface layer of the studied sample. Assuming the values of the D-parameter for diamond and graphite linear interpolation is performed, and for the given D, %sp^2^ is obtained.

### 3.5. Morphological Studies

To determine the morphology of the obtained carbons, images of the powder samples were taken using a high-resolution Quanta 3D FEG scanning electron microscope from FEI Company (Hillsboro, OR, USA) at an acceleration voltage of 5 kV. Due to electrostatic charging during the analysis, the sample was covered with a thin layer of Pd/Au.

### 3.6. Porous Structure Determination

The porous structure of the carbon sorbents was characterized by means of nitrogen adsorption–desorption measurements using an adsorption analyzer, ASAP 2420 Micromeritics Inc. (Norcross, GA, USA). The determination was based on the measurements of adsorption and desorption of nitrogen on the surface of the studied sorbents while cooling it to liquid nitrogen. The specific surface areas were calculated by the Brunauer–Emmett–Teller (BET) method, assuming that the area of a single nitrogen molecule is 0.162 nm^2^. The pore volumes and pore size distributions were determined by the Barrett–Joyner–Halenda (BJH) method. The sample degassing temperature was 150 °C.

### 3.7. Adsorption/Desorption Experiments of Ciprofloxacin

For the ciprofloxacin (CF) adsorption experiments, ciprofloxacin hydrochloride monohydrate was taken as the adsorbate. Ionic strength was adjusted with NaCl (ITES, p.a., 99%) to 0.1 M.

#### 3.7.1. pH Dependence

The dependence of the uptake on pH was investigated in the pH range from 2 to 7.5. All adsorption experiments were performed in a static mode at ambient temperature. The initial CF concentration was 10 mg⋅L^−1^, and the adsorbent dose of 0.01 g was added into 10 mL of CF solution for 24 h.

#### 3.7.2. Kinetic Experiment

In order to study the effect of contact time, 0.01 g of the test sample was poured with 10 mL of 10 mg⋅L^−1^ (pH~6.67) CF solution and the contact time varied for each experiment in the range of 1–24 h.

#### 3.7.3. Adsorption Equilibrium

The dependence of adsorption on the initial concentration of CF was studied under the following conditions: dosage of sorbent = 1 g⋅L^−1^; concentration of CF from 10 to 300 mg⋅L^−1^; pH~6.67–7.2; contact time 24 h.

After the adsorption process in all studies (pH, kinetics, isotherms), the content of each tube was filtered through the ashless filter paper ‘Fisher scientific’. The concentration of the adsorbate in the initial and residual solutions was determined by a Helios Gamma UV-Vis spectrophotometer (Thermo electron corporation, UK), measuring the antibiotic absorbance at 275 nm. The adsorption capacity *A* (mg⋅g^−1^) of the adsorbent was calculated from Equation (1):

(1) $$A=\frac{{(C}_{0}-C_{eq\;})V}{m}$$

where *C*_0_ and *C_eq_* are the initial and equilibrium concentrations, respectively (mg⋅L^−1^), *V* is the volume of the solution (L), and *m* is the mass of the adsorbent (g).

#### 3.7.4. Desorption Studies

To facilitate the regeneration and reuse of adsorbent materials, the desorption process must be investigated to ensure efficient removal of contaminants. Selecting an appropriate eluent is a critical step, as it affects directly the effectiveness of pollutant extraction from the sample matrix. Based on the contaminant properties and relevant literature, a series of eluents was selected for further evaluation to optimize desorption efficiency.

Samples loaded with ciprofloxacin were used for this experiment; a measure of 10 mg of each CF-containing sample was treated with 10 mL of each eluent and shaken (Orbital Shaker OS-20, 200 rpm) for 24 h. Then, the solution was separated by filtration, and its concentration was determined using a UV-Vis spectrophotometer.

The obtained results were calculated using Equation (2). The desorption efficiency (%) of CF was calculated using

(2) $$D=\frac{A_{des}}{A}\times 100$$

where *D* (%) is the CF desorption efficiency (%) and *A_des_* and *A* are the CF desorption and adsorption capacities, respectively.

The tested eluents included distilled water, ethanol, 0.1 M NaOH, 0.1 M HCl, (70:30) EtOH: 0.1 M NaOH, and (70:30) EtOH: 0.1 M HCl.

The adsorption and desorption of ciprofloxacin for the samples were performed as described above. Adsorption was conducted from an aqueous ciprofloxacin solution with a concentration of 200 mg L^−1^ for 24 h under the static conditions, using 10 mg of the sample and 10 mL of the solution. After adsorption, the ciprofloxacin-loaded sample was rinsed with 10 mL of water, air-dried, and used for desorption. After desorption, the sample was rinsed with water until a neutral pH was reached, air-dried, and reused for the next adsorption cycle.

To evaluate the physical stability of the adsorbent during reuse, the dry mass was measured after each desorption step across multiple adsorption–desorption cycles. The stability of adsorbent mass was calculated as

(3) $$Mass\;of\;adsorbent,\;\%=\frac{m_{0}-m}{m_{0}}\times 100$$

where m_0_ (g) is the initial mass of the adsorbent before the first adsorption cycle and m (g) is the dry mass of the adsorbent after each desorption cycle.

## 4. Conclusions

Coffee grounds impregnated with phosphoric acid are ideal for obtaining carbon sorbents via microwave carbonization. The reactor conditions allow for the production of sorbents with a well-developed porous structure and interesting surface functional groups after just 5 min of irradiation.

Plasma treatment causes minor changes in the porous structure, but it is largely effective in restructuring the surface chemistry, as demonstrated by the XPS analysis.

The XPS analysis revealed that the surface chemistry of coffee-based activated carbons is largely dependent on both the duration of microwave irradiation and the subsequent cold plasma treatment. The C=C sp^2^ concentration in the samples after plasma treatment increased. For the initial samples (C2–C10), it was in the range of 41.2–60.2 wt.%, while after activation, it was 56.2–75.7 wt.%. An increase in oxygen groups (C=O) and changes in the concentrations of amine/amide and quaternary N groups were also observed. The ability to tailor the specific functionalities of carbon, oxygen, nitrogen, and phosphorus provides a versatile platform for the design of low-cost heteroatom-doped carbon materials.

Sorption studies of the antibiotic ciprofloxacin (CF) showed that, in particular, both samples obtained after 10 min of carbonization (C10) and subsequent plasma activation (C10_act_) show the highest sorption capacity in relation to CF (92.4 and 120.6 mg g^−1^, respectively). It is worth emphasizing that C10_ac_ can be effectively used as an adsorbent for eight cycles, with regeneration and reuse for ciprofloxacin adsorption.

These materials can have broader applications that are not taken into account in this paper, including energy storage, catalysis, sensors, and environmental protection.

## Figures and Tables

**Table 1:** Porous structure of coffee-based activated carbons.

Sample	Specific Surface Area, m^2^ g^−1^	Total Pore Volume, cm^3^ g^−1^	Micropore Volume, cm^3^ g^−1^	Mesopore Volume, cm^3^ g^−1^
C2	26	0.11	0 (0%)	0.11 (100%)
C5	325	0.47	0.07 (15%)	0.40 (85%)
C10	482	0.65	0.11 (17%)	0.53 (83%)
C2_act_	44	0.16	0 (0%)	0.15 (100%)
C5_act_	425	0.55	0.10 (18%)	0.45 (82%)
C10_act_	601	0.69	0.15 (22%)	0.54 (78%)

**Table 2:** Data from the SEM-EDS microanalysis of coffee-based activated carbons.

Sample	Element, wt.%			
	C	N	O	P
C2	62.54	5.11	28.72	3.63
C5	75.50	4.95	15.31	4.24
C10	77.37	4.38	13.75	4.50
C2_act_	62.81	5.03	29.42	2.74
C5_act_	75.15	5.02	16.57	3.26
C10_act_	77.79	4.48	13.88	3.85

**Table 3:** C (1s) data from the XPS of coffee-based activated carbons.

Sample	Name, %At. Conc.					
	C=C sp^2^	C–C/C–H	C–OH	C=O	COOR	π-π* Shake-Up
C2	41.2	25.5	17.0	6.7	4.2	5.4
C5	47.7	22.3	15.1	5.8	3.5	6.1
C10	60.2	13.8	11.3	5.0	3.3	6.4
C2_act_	56.2	12.3	9.3	11.3	4.3	6.6
C5_act_	70.4	9.8	6.0	5.4	2.4	5.5
C10_act_	75.7	7.0	5.9	2.9	2.2	6.1

**Table 4:** O (1s) data from the XPS of coffee-based activated carbons.

Sample	Name, %At. Conc.		
	O=C/O=C–O–R	C–OH	Car–OH/O–C/O=C–O–R
C2	18.1	60.7	21.3
C5	25.2	59.3	15.4
C10	38.8	48.9	12.3
C2_act_	20.4	49.1	30.5
C5_act_	32.1	44.1	18.7
C10_act_	42.0	41.1	16.9

**Table 5:** N (1s) data from the XPS of coffee-based activated carbons.

Sample	Name, %At. Conc.				
	Pyridinic N	Amines/Amides	Pyrrolic N	Quaternary N	N–O (Oxidized N)
C2	13.8	57.3	21.3	0.3	7.2
C5	23.2	38.4	20.8	11.6	6.0
C10	25.9	36.2	16.1	16.3	5.4
C2_act_	13.9	15.3	38.1	22.6	10.2
C5_act_	22.4	32.1	27.8	9.8	7.9
C10_act_	20.1	33.0	28.5	9.7	8.7

**Table 6:** Kinetic sorption parameters obtained using the pseudo-first- and pseudo-second-order models for CF adsorption.

Sample		Pseudo-First-Order			Pseudo-Second-Order		
	q_e_ (exp) (mg g^−1^)	q_e_ (cal) (mg g^−1^)	k_1_ (min^−1^)	R^2^	q_e_ (cal) (mg g^−1^)	k_2_ (g mg^−1^h^−1^)	R^2^
**C2**	1.12	9.57	0.104	0.867	1.74	0.156	**0.930**
**C5**	7.94	0.59	0.331	0.049	8.33	0.197	**0.994**
**C10**	9.19	0.70	0.141	0.047	10.26	0.233	**0.997**
**C10_act_**	9.50	0.68	0.101	0.648	10.32	0.251	**0.998**

**Table 7:** Parameters of CF adsorption using the Langmuir and Freundlich isotherm models.

Sample	A, mg g^−1^	Langmuir Model			Freundlich Model		
		q_max_ (mg g^−1^)	K_L_ (L mg^−1^)	R^2^	1/*n*	K_F_ (mg g^−1^)	R^2^
**C2**	20.2	30.3	0.0036	**0.999**	0.95	7.27	0.972
**C5**	71.2	78.1	0.045	**0.999**	0.5	5.71	**0.908**
**C10**	92.4	65.8	0.199	**0.968**	0.45	10.25	**0.956**
**C2_act_**	42.6	81.3	0.003	**0.997**	1.01	3.95	**0.945**
**C5_act_**	102.6	87.7	0.085	**0.994**	0.53	7.68	**0.977**
**C10_act_**	120.6	138.9	0.141	**0.999**	0.45	18.35	**0.849**

**Table 8:** Comparative study of adsorption capacities of different adsorbents for ciprofloxacin removal.

Adsorbent	Maximum Adsorption Capacity, q_max_, mg g^−1^	Reference
Activated carbon from mangosteen peel	29.78	[33]
Biochar derived from co-pyrolysis of sewage sludge and bamboo waste	62.48	[34]
CO_2_ activated biochar-clay mineral composite prepared at 350 °C	50.32	[35]
Iron and nitrogen co-doped biochar (Fe/N-BC)	46.45	[36]
Powdered activated commercial carbon (PAC)	71.9	[37]
Modified bamboo biochar (MBC)	78.43	[38]
Chitosan modified Fe pretreated biochar (CS-FBC)	76.72	[39]
Biochar from rice straw (RBC) modified by KmnO_4_ and NaOH (Mn/Na-RBC)	32.25	[40]
C10_act_	**138.9**	**Present study**

## Data Availability

Dataset available on request from the authors.

## Supplementary Materials

The following supporting information can be downloaded at https://www.mdpi.com/article/10.3390/molecules30204123/s1: Figure S1: C (1s) XPS spectra of coffee-based activated carbons; Figure S2: O (1s) XPS spectra of coffee-based activated carbons; Figure S3: N (1s) XPS spectra of coffee-based activated carbons.
