# Supplementary material for: Microwave-Assisted Preparation of Coffee-Based Activated Carbons: Characteristics, Properties, and Potential Application as Adsorbents for Water Purification
Source: Molecules. 2025 Oct 17;30(20):4123. doi: 10.3390/molecules30204123 (PMC12566009; doi:10.3390/molecules30204123)
Supplement: Supplementary file 1 [file molecules-30-04123-s001.zip › molecules-3930256-SM.pdf]

molecules

MDPI

Article

Microwave-Assisted Preparation of Coffee-Based Activated Carbons: Characteristics, Properties, and Potential Application as Adsorbents for Water Purification

Przemysław Pączkowski [1], Viktoriia Kyshkarova [2], Viktor Nikolenko [3], Oksana Arkhipenko [3,4], Inna Melnyk [2,5] and Barbara Gawdzik [1,*]

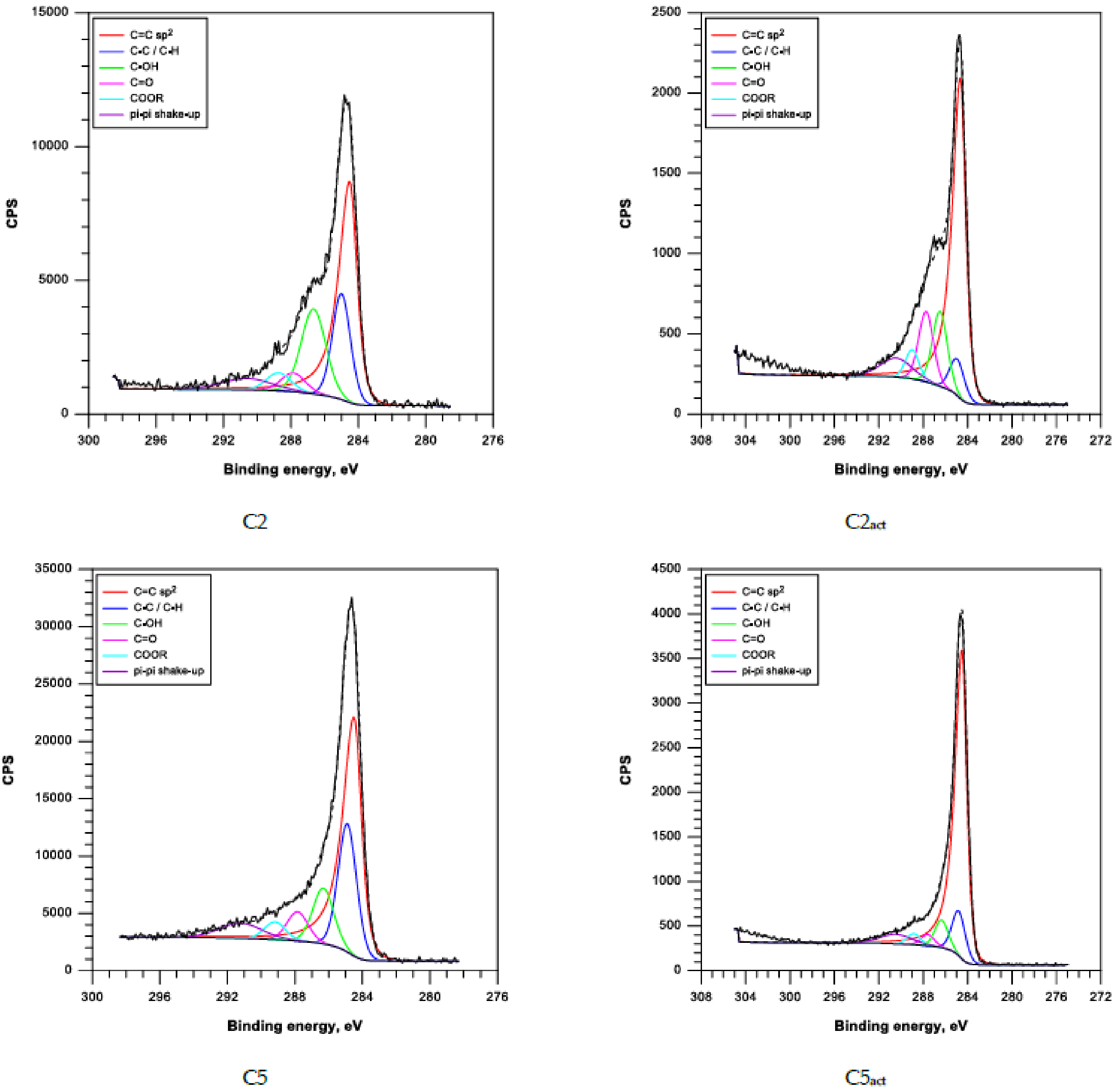

Figure S1. C (1s) XPS spectra of coffee-based activated carbons.

 https://doi.org/10.3390/molecules30204123

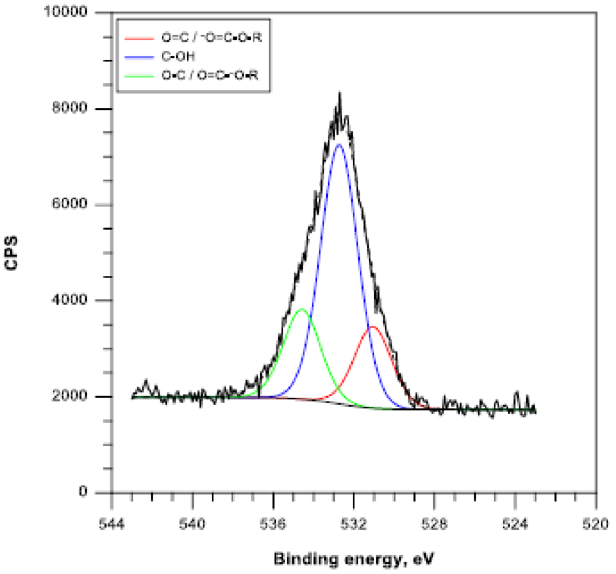

C2

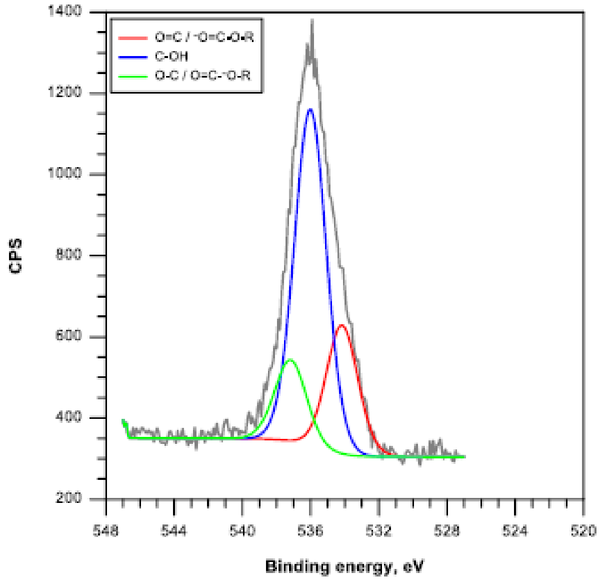

C2act

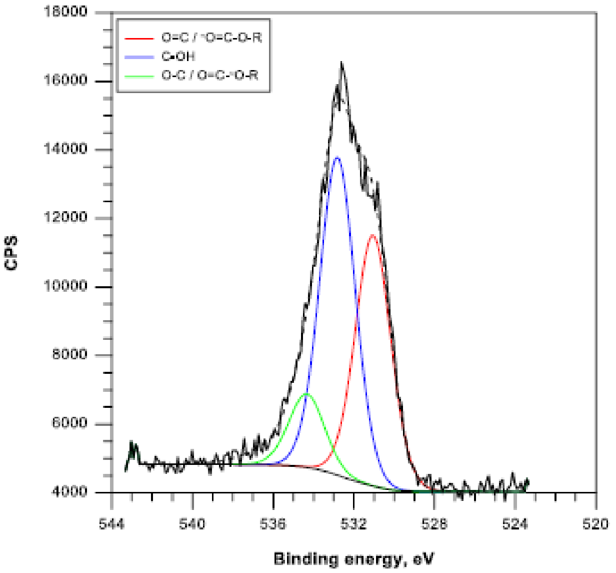

C5

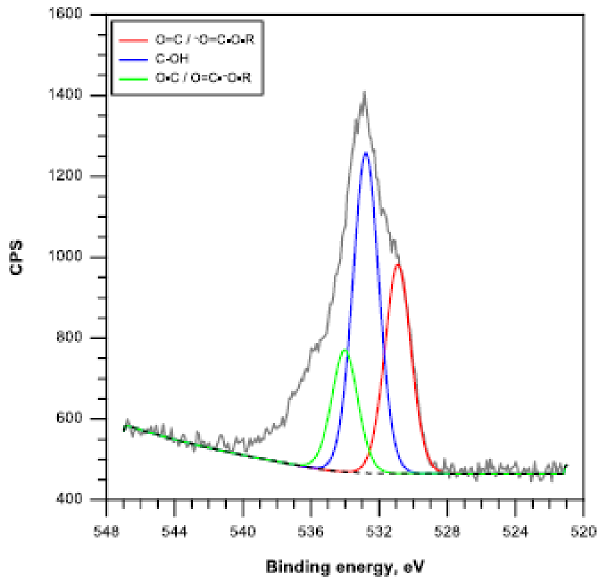

C5act

Figure S2. O (1s) XPS spectra of coffee-based activated carbons.

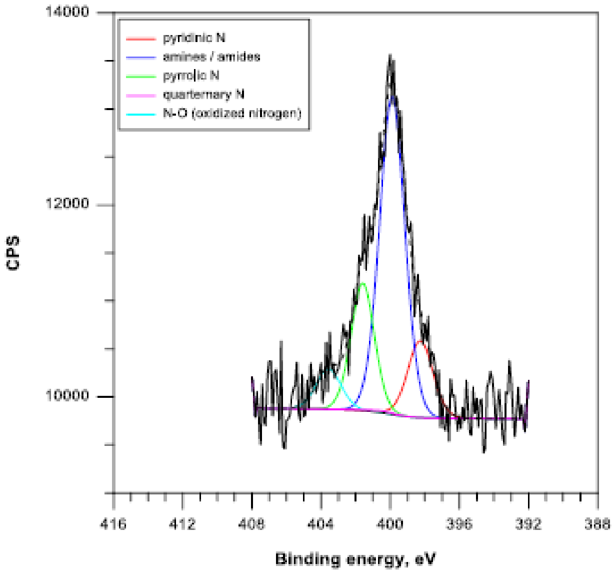

C2

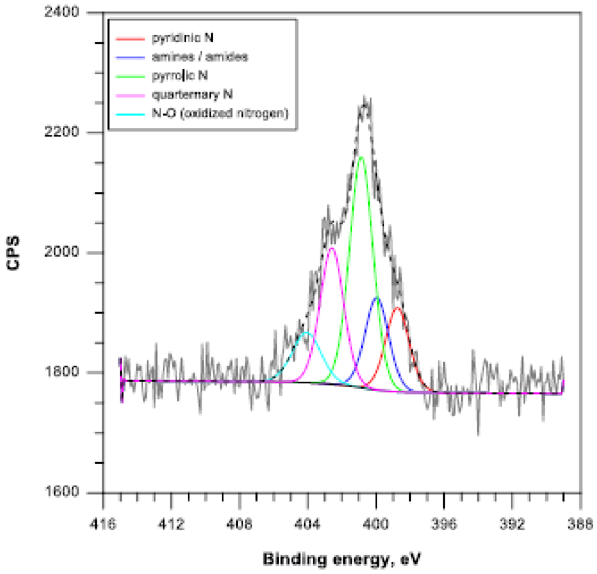

C2act

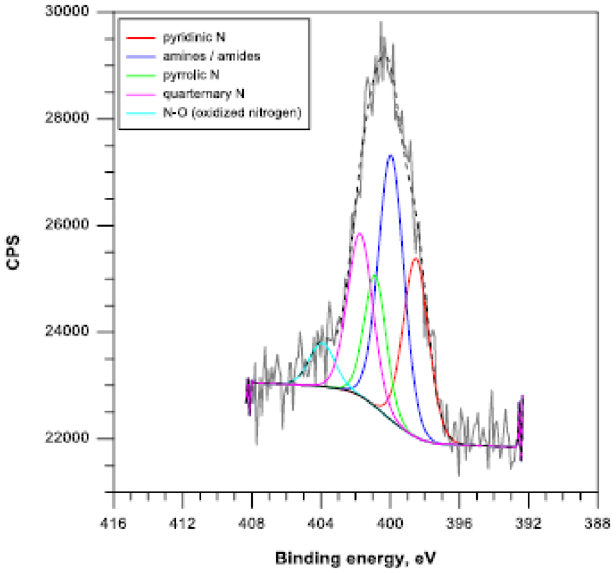

C5

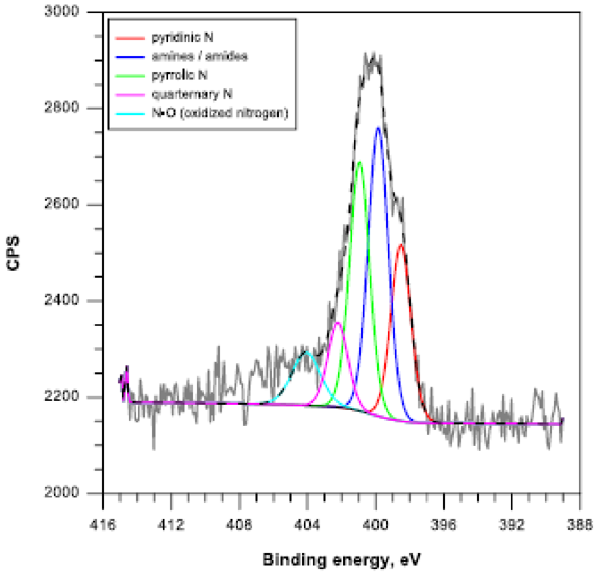

C5act

Figure S3. N (1s) XPS spectra of coffee-based activated carbons.

Disclaimer/Publisher's Note: The statements, opinions and data contained in all publications are solely those of the individual author(s) and contributor(s) and not of MDPI and/or the editor(s). MDPI and/or the editor(s) disclaim responsibility for any injury to people or property resulting from any ideas, methods, instructions or products referred to in the content.
